# The hypothesis of sympatric speciation as the dominant generator of endemism in a global hotspot of biodiversity

**DOI:** 10.1002/ece3.1761

**Published:** 2015-10-26

**Authors:** Markus Gastauer, Amílcar Walter Saporetti‐Junior, Luiz Fernando Silva Magnago, Jeannine Cavender‐Bares, João Augusto Alves Meira‐Neto

**Affiliations:** ^1^ Center of Environmental Research Floresta‐Escola Av. Prof. Mario Palmério 1000 38200‐000 Frutal MG Brazil; ^2^ Laboratory of Ecology and Evolution of Plants Universidade Federal de Viçosa 36570‐000 Viçosa MG Brazil; ^3^ Department of Ecology, Evolution and Behavior University of Minnesota St. Paul Minnesota 55108

**Keywords:** Brazilian Atlantic Forest, dispersal limitation, phylogenetic community structure, similarity, spatial phylogenetic turnover, species formation

## Abstract

Allopatric or sympatric speciation influence the degree to which closely related species coexist in different manners, altering the patterns of phylogenetic structure and turnover among and between communities. The objective of this study was to examine whether phylogenetic community structure and turnover in the Brazilian Atlantic Forest permit conclusions about the dominant process for the formation of extant angiosperm richness of tree species. Therefore, we analyzed phylogenetic community structure (MPD, MNTD) as well as taxonomic (Jaccard similarity) and phylogenetic turnover (betaMPD, betaMNTD) among and between 49 tree communities distributed among three different habitat types. Mean annual precipitation and mean annual temperature in each survey area were estimated. Phylogenetic community structure does not differ between habitat types, although MPD reduces with mean annual temperature. Jaccard similarity decreases and betaMNTD increases with spatial distance and environmental differences between study sites. Spatial distance explains the largest portions of variance in the data, indicating dispersal limitation and the spatial aggregation of recently formed taxa, as betaMNTD is related to more recent evolutionary events. betaMPD, that is related to deep evolutionary splits, shows no spatial or environmental pattern, indicating that older clades are equally distributed across the Brazilian Atlantic Forest. While similarity pattern indicates dispersal limitations, the spatial turnover of betaMNTD is consistent with a high degree of sympatric speciation generating extant diversity and endemism in the Brazilian Atlantic Forest. More comprehensive approaches are necessary to reduce spatial sampling bias, uncertainties regarding angiosperm diversification patterns and confirm sympatric speciation as the dominant generator for the formation of extant species diversity in the Brazilian Atlantic Forest.

## Introduction

The magnificent biodiversity of tropical biomes has been investigated for a long time; biome size, persistence and trophic interactions are considered as the motor of evolution to explain extant species richness (Terborgh 1973; Rosenzweig 1995; Dyer 2008; Fine et al. 2008). Elevated speciation rates cause accumulation of species (Mittelbach et al. 2007; Donoghue 2008; Moreau et al. 2006; Moreau and Bell 2013; Rosindell et al. 2011, 2012; Rolland et al. 2014), although the type of speciation itself – allopatric or sympatric – is rarely discussed in the literature (Hoorn et al. 2011; Naka et al. 2012; Smith et al. 2014). While allopatric speciation is the most refered as biodiversity generator by theories such as the Refugia Hypothesis (Haffer 1967, 1969; Haffer and Prance 2001; Carnaval and Moritz 2008), evidence is increasing that species also evolve in sympatry (MacArthur and Wilson 1963, 1967; Gentry 1986; Grant and Grant 2002; Spironello and Brooks 2003; Barluenga et al. 2006; Losos et al. 2006; Bowen et al. 2013).

**Figure 1:**
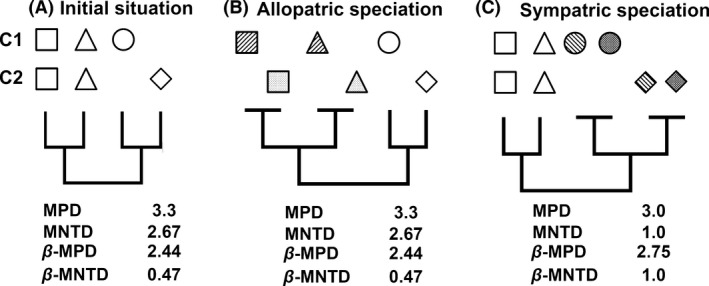
Influence of different speciation patterns in two hypothetical communities C1 and C2 on phylogenetic community structure and phylogenetic turnover. (A) shows the initial situation with four species distributed within both communities with hypothetic phylogenetic relationships as illustrated in the embedded tree. (B) shows the outcomes of allopatric, and (C) of sympatric speciation (MPD is mean phylogenetic distance, MNTD is mean nearest taxon distance, and betaMPD and betaMNTD are MPD and MNTD between pairs of species from Northern and Southern parts of the biome). Identical symbols with different hatchings indicate sister species. Due to small number of species in this hypothetical example, phylogenetic community structure and phylogenetic turnover in the allopatric speciation scenario (B) do not change from the initial situation. Further, species‐richer examples show that MPD and MNTD increase, while betaMPD and betaMNTD reduce.

Different speciation processes are expected to leave different traces in extant species’ distribution ranges, thus influencing phylogenetic community structure and phylogenetic turnover between sites (Barraclough and Vogler 2000; Graham and Fine 2008; Warren et al. 2014) (Fig. 1). Allopatric speciation tends to create distinct distributional ranges for closely related species (Savolainen et al. 2006; Kamilar et al. 2009). Such occupation of distinct geographic ranges by sister species results in phylogenetic evenness and low phylogenetic turnover between spatially distant communities (Hardy and Senterre 2007) (Fig. 1B). Sympatric speciation is supposed to cause sister species to coexist in spatial aggregations (e.g., Anacker and Strauss 2014). Therefore, a scenario dominated by sympatric speciation is expected to cause phylogenetic clustering as well as phylogenetic turnover that increases with spatial distance between communities (Johnson and Stinchcombe 2007; Graham and Fine 2008) (Fig. 1C).

These opposite evolutionary processes are not the only phenomena that shape extant species’ distributions. Secondary contact of allopatric evolved species may impede the recognition of evolutionary processes from actual species distributions and patterns of phylogenetic community structure and turnover (Losos and Glor 2003). Furthermore, contemporary processes such as habitat specialization cause similar species sharing functional traits to coexist (Simberloff 1970; van der Valk 1981; Weiher and Keddy 1995), whereas overlap of ecological niches limits coexistence due to interspecific competition and further density‐dependent interactions (Gause 1934; Hardin 1960; Diamond 1975). Therefore, these and further ecological patterns influence phylogenetic community structure and turnover (Hardy and Senterre 2007; Cavender‐Bares et al. 2009) and should be outlined before conclusions regarding biogeographic patterns may be drawn.

The Brazilian Atlantic Forest is a diverse and well‐studied ecosystem comprising 27 latitudinal degrees. It includes areas with altitudes near sea level and other areas at altitudes of nearly 3000 m; certain parts of the ecosystem are arid, whereas others experience 3600 mm of rain per year (Stehmann et al. 2009). Within it, environmental heterogeneity has resulted in the presence of three principal types Seasonal Semideciduous Forests, Evergreen Mixed Forests, and Evergreen Dense Forests (Veloso et al. 1991). Recent census compiled a list of about 14,000 angiosperm species native to the Atlantic Forest (Forzza et al. 2014), of which around half are endemic (Stehmann et al. 2009; Critical Ecosystems Partnership Fund 2014).

Although fundamental questions concerning the factors that permitted this diverse ecosystem to evolve have engaged ecologists for decades (i.e., Ab'Saber 1977; Oliveira‐Filho and Fontes 2000; Oliveira et al. 2014), they are still controversial (Gastauer and Meira‐Neto 2014). In this study, we aimed to outline whether sympatric or allopatric speciation was the dominant processes in the generation of 1548 angiosperms tree species occurring within 49 communities in the Brazilian Atlantic Forest. As distribution ranges resulting from different diversification processes are distorted by environmental filtering, interspecific competition, and further contemporary factors, our goal was to disentangle their relative importance in this hyperdiverse forest region by analysis of phylogenetic community structure, taxonomic, and phylogenetic turnover among and between communities.

## Methods

### Database

Within the Atlantic Forest, we selected 49 surveys of tree communities from Seasonal Semideciduous (27 communities), Evergreen Mixed (5), and Evergreen Dense Forests (17). These data were from the literature or from our unpublished database form the Laboratory of Ecology and Evolution of Plants from the Universidade Federal de Viçosa, Brazil (Appendix S1 in Supporting Information). All surveys from the literature include either geographic coordinates, a map or an aerial image that allows the inference of the exact location of the community. Abundance data on the surveyed species are available, each sampled area was at least 0.5 ha, and comprised more than 400 sampled individuals. Finally, more than 98% of angiosperm species and more than 95% of angiosperm individuals from the community were identified, at least at the genus level.

Species lists from all references were spell‐checked, and systematic information was updated using the database of the Missouri Botanical Garden (2013). For that, we used the Taxonomic Name Resolution Service (Boyle et al. 2013). Species not found in the Missouri database were classified according to Forzza et al. (2014).

Because the surveys were taken from the literature, the nomenclature of taxa that were not identified at the species level was not consistent. A taxon from a certain community identified at the genus or family level only may correspond to (1) a fully identified species from another community, (2) a taxon from another community that was not identified at the species level, or (3) a new taxon not previously represented in the database. To address this problem, we conducted two analyses: first, taxa not identified to species level from the same genus/family from different studies were pooled into the same taxon (small dataset); second, we considered each taxa that was not identified at the species level from each survey as a separate taxon (large dataset). The small dataset contains 1548 taxa belonging to 437 genera and 96 families; the large dataset is composed of 1869 taxa. Incomplete identification of some taxa in the database would not influence the results reached in the study if the slope and coefficient of correlation between outcomes generated from both datasets are 1.

Communities were grouped according to their habitat type Seasonal Semideciduous, Evergreen Dense, or Evergreen Mixed Forest according to Veloso et al. (1991). Average annual precipitation and mean annual temperature in each survey area were estimated from the area's geographic position using New_LocClim (Grieser et al. 2006). Geographic distances in kilometers between sampled communities were computed from coordinates; furthermore, we computed the differences in precipitation regime (measured in mm, MAP) and mean annual temperature (°C, MAT) between all pairs of communities.

### Phylogenetic community structure

We used the mean pairwise distance (MPD) and the mean nearest taxon distance (MNTD) between different taxa as indices for phylogenetic community structure because they are not influenced by metacommunity size (Webb et al. 2002). The larger MPD or MNTD values for a community, the more it is phylogenetically overdispersed. The smaller the values, the stronger is its phylogenetic clustering. All indices were computed with the software Phylocom‐4.2 (Webb et al. 2002). Whereas MPD indicates tendencies over the whole phylogenetic tree, including deep evolutionary splits, the MNTD emphasizes structures occurring toward the tip of the phylogenetic tree regarding more recent evolutionary events (Cavender‐Bares et al. 2006).

**Figure 2:**
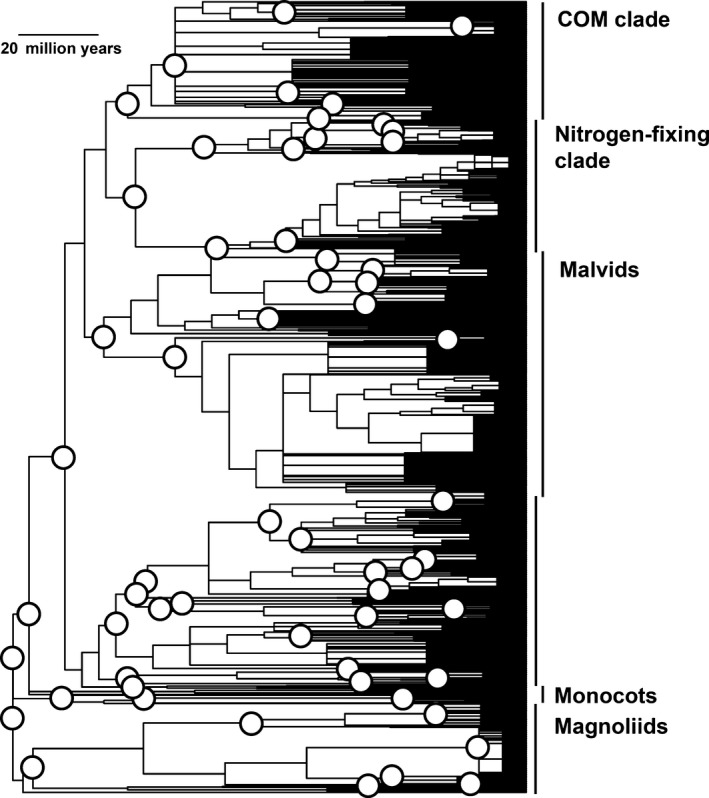
Hypothesized phylogenetic relationships among woody angiosperms from small dataset. Circles indicate nodes dated by divergence times reported by Bell et al. (2010). Undated nodes were spaced evenly between dated nodes.

For computation of indices, megatree R20120829.new (Gastauer and Meira‐Neto unpubl. ms) was pruned to all tree taxa from the database (1548 in the small dataset and 1869 in the large dataset) using the phylomatic function from Phylocom 4.2 (Webb and Donoghue 2005). The resulting phylogenetic community trees were calibrated using the bladj algorithm of the Phylocom package and age estimates from Bell et al. (2010) as suggested by Gastauer and Meira‐Neto (unpubl. ms) (Fig. 2).

### Taxonomic and phylogenetic turnover

We computed the Jaccard similarity between all possible pairs of tree communities using EstimateS 8.0 (Colwell and Coddington 1994). The larger the Jaccard similarity, the lower the taxonomic beta‐diversity between communities.

Phylobetadiversity, also known as phylogenetic turnover, was computed as betaMPD and betaMNTD between different taxa from each pair of communities using the Phylocom 4.2 package (Fine and Kembel 2011). The larger the betaMPD or betaMNTD for a pair of communities, the larger is the phylogenetic turnover between them.

To identify pairs of communities characterized by a low phylogenetic turnover, we computed the betaNRI (Net Relatedness Index) and the betaNTI (Nearest Taxon Index) as the negative standardized effect size of the betaMPD and the betaMNTD as proposed by Webb et al. (2002) using the unconstrained null model (Kembel and Hubbell 2006). For this analysis, 10,000 randomizations in which species identities were shuffled within surveys were conducted. Phylogenetic turnover was considered low if the indices had positive values. In contrast, negative values indicated high phylogenetic turnover. Significance for individual values was indicated if betaNRI or betaNTI was higher than 1.96 (low phylogenetic turnover) or lower than −1.96 (high phylogenetic turnover). Pairs of communities with significantly low turnover were plotted on a map to identify spatial aggregations in which closely related species co‐occur.

### Phylogenetic resolution

The resolution of the phylogenetic community tree might influence the outcomes and the interpretation of phylogenetic community structure and phylobetadiversity (Swenson 2009). Because the phylomatic command of the Phylocom package treats species belonging to the same genus or family as a polytomy, the number of unresolved taxa (1119 or 71.5% for the small dataset and 1440 or 80.3% for the large dataset) is high. To examine the influence of this lack of phylogenetic resolution, we randomly transformed 10,000 times all polytomies from the trees containing the small dataset into a series of dichotomies using the multi2di algorithm from the ape extension (Paradis et al. 2014) of R Environment, version 3.1.0 (R Development Core Team 2014). These completely resolved trees were calibrated by the bladj algorithm using age estimates from Bell et al. (2010); then, the indices MPD, MNTD, betaMPD, and betaMNTD were calculated as described above. Lacking phylogenetic resolution would not influence the results reached in this study if the slope and coefficient of correlation between outcomes computed from both fully resolved and unresolved communities are 1.

### Data analysis

The data distribution of phylogenetic community structure, taxonomic and phylogenetic turnover was assessed for normality by a Shapiro–Wilk test. Because phylogenetic community structure and turnover were not distributed normally, the original data were transformed by common logarithm to achieve normal error distributions.

Differences in phylogenetic community structure among different habitats were checked using a one‐way ANOVA. To outline the influence of the environment variables mean annual temperature as well as mean annual precipitation on the phylogenetic community structure (log‐transformed MPD and MNTD values), we built general linearized models (GLM) without any type of interactions (Zuur et al. 2009).

The GLM were built using the “glm” command in the R Environment. We used the dredge function from the “MuMIn” package (Bartón 2014) in R Environment, version 3.1.0 (R Development Core Team 2014) to test all possible combinations of the variables included in the global models as well as the null model. To determine which combinations of explanatory variables were the most parsimonious, we used an information‐theoretic approach based on the Akaike information criterion of second order (AICc); thus, the best model was indicated by the AICc lower value (Burnham et al. 2011).

Simple and partial Mantel tests were performed to evaluate the contributions of log‐transformed spatial distance or environmental differences in mean annual precipitation (MAP) and in mean annual temperature (MAT) between pairs of communities on Jaccard similarity or phylogenetic turnover (log‐transformed betaMPD and betaMNTD values). For that, we applied “ecodist” package (Goslee and Urban 2015) in R Environment, version 3.1.0 (R Development Core Team 2014). The simple correlation of taxonomic and phylogenetic turnover with spatial distance, MAT or MAP via Mantel test seeks for spatial and environmental pattern of taxonomic and phylogenetic turnover. Partial Mantel tests were carried out to compare the correlation between two matrices, for example, Jaccard similarity and spatial distance, but taking into account a third one, for example, MAT or MAP. All possible combinations were tested to meet a hierarchy of correlations.

To outline the importance of spatial distance, MAT and MAP on taxonomic similarity and phylogenetic turnover and justify spatial classification of communities in spatial aggregations, we fitted multiple membership models using lmer from the lme4 package (Bolker 2015) including a further categorical variable indicating if surveys are from the same spatial aggregation or not. We built mixed general linearized mixed models (GLM) without any type of interactions, codifying names of both communities related to the similarity or phylogenetic turnover measure as random variables. Then, we used the dredge function to test all possible combinations of the variables included in the global models as well as the null model; thus, the best model was indicated by the AICc lower value.

## Results

### Phylogenetic community structure

**Figure 3:**
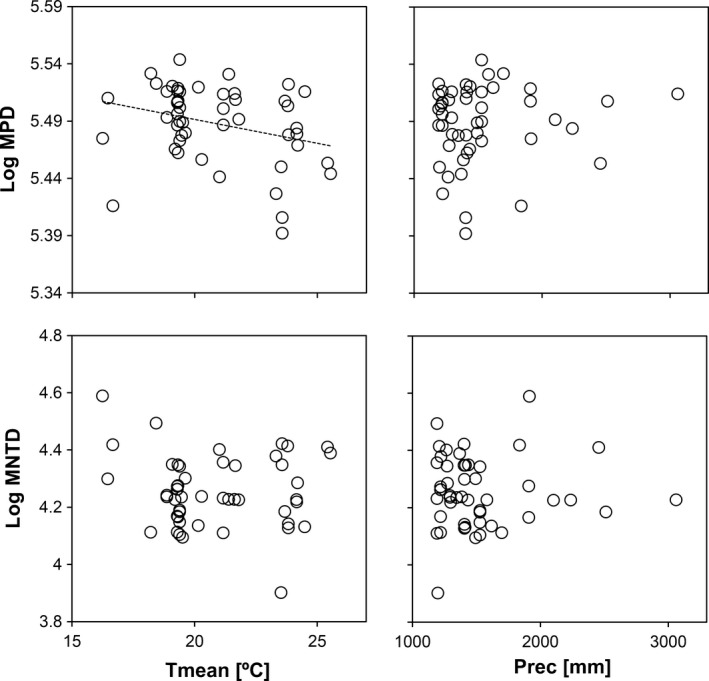
Best GLM indicated by trend lines (dashed: *P* < 0.1) explaining log‐transformed mean pairwise distance (log MPD) and mean nearest taxon distance (log MNTD) in terms of mean annual temperature (Tmean) and precipitation (Prec).

The log‐transformed MNTD values of the analyzed tree communities show no relation with mean annual precipitation or mean annual temperature (Fig. 3). On contrast, the log‐transformed MPD reduces with mean annual temperature (*P* < 0.1, Fig. 3). Log‐transformed MPD values do not vary among different habitat types, while MNTD of Evergreen Mixed Forests is significantly higher than that from Evergreen Dense Forests (Appendix S2).

Strong correlations with the outcomes from the large dataset indicate that the results are not constrained by incomplete identification (Table 1 from Appendix S3). With slope values of approximately 1 and *r* ^2^ values above 0.95, the MPDs of the fully resolved phylogenies are well predicted by the unresolved phylogeny obtained from the phylomatic procedure. The MNTD values from the fully resolved phylogenies do not correlate to the same extent with the unresolved phylogeny (Table 2 from Appendix S3). Accordingly, further enhancements of the phylogenetic resolution might alter these findings slightly.

### Taxonomic and phylogenetic turnover

Simple Mantel tests show that Jaccard similarity reduces while phylogenetic turnover, measured by betaMNTD, increases with spatial distance and environmental differences between communities (Table 1). On contrast, betaMPD does not show significant relations with spatial distance or environmental differences between communities.

**Table 1:** Pearson correlation coefficients of simple and partial Mantel tests between taxonomic and log‐transformed phylogenetic turnover and spatial distance, differences in mean annual temperature (MAT) or mean annual precipitation (MAP) between study sites. *** indicates significance level of 0.001, ** is *P* < 0.01, and * is *P* < 0.05

Variable 1	Variable 2	Simple Mantel test	Partial Mantel tests			
			Spatial distance	MAT	MAP	All
J	Spatial distance	−0.730***	–	−0.655***	−0.726***	−0.623***
	MAT	−0.560***	−0.323**	–	−0.554***	−0.320**
	MAP	−0.187*	−0.154*	−0.162*	−	−0.147*
betaMNTD	Spatial distance	0.613***	−	0.473**	0.610***	0.472***
	MAT	0.526***	0.316**	–	0.522***	0.314**
	MAP	0.071	0.001	0.024	−	−0.014
betaMPD	Spatial distance	−0.128	–	−0.143	−0.145	−0.154
	MAT	−0.009	0.065	–	−0.195	0.059
	MAP	0.127	0.144	0.121	–	0.141

Correlations between Jaccard similarity and betaMNTD with spatial distance were stronger than that with MAT and MAP. Taking into account further matrices (i.e., one or both remaining environmental differences and/or spatial distance), spatial distance explains highest percentage of Jaccard similarity as well as betaMNTD (Table 1). On contrast, the log‐transformed betaMPD shows no significant correlation with spatial distance, MAT or MAP (Table 1).

The Jaccard similarity indicates a higher similarity between pairs of surveys from the same habitat than between pairs of surveys from different habitats (Table 2). betaMNTD among communities from SSF and EMF is lower than among EDF and between communities from different habitats. betaMPD shows no significant differentiation among or between different habitat types (Table 2).

**Table 2:** Mean values of similarity and phylogenetic turnover and their standard deviations between pairs of surveys from different habitat types and different spatial aggregations are shown in Figure 4. Different superscript letters indicate statistically significant differences according to a one‐way ANOVA (*P* < 0.05). betaMPD is the mean pairwise distance from a pair of communities, and betaMNTD is the mean nearest neighbor distance from a pair of communities

Comparison	Jaccard similarity	betaMNTD	betaMPD
SSF‐SSF	0.120 ± 0.084^b^	63.04 ± 16.14^d^	222.17 ± 1.92^a^
SSF‐EDF	0.064 ± 0.041^d^	71.10 ± 16.25^c^	222.66 ± 2.52^a^
SSF‐EMF	0.075 ± 0.045^c^	73.84 ± 16.42^b^	222.65 ± 1.97^a^
EDF‐EDF	0.076 ± 0.065^c^	70.48 ± 21.86^c^	222.70 ± 2.59^a^
EDF‐EMF	0.045 ± 0.036^e^	82.42 ± 17.33^a^	222.71 ± 1.64^a^
EMF‐EMF	0.184 ± 0.083^a^	53.94 ± 9.57^d^	220.55 ± 2.96^a^
Do‐Do	0.181 ± 0.077^b^	43.67 ± 9.61^g^	221.51 ± 1.63^c^
Do‐NE	0.061 ± 0.033^e^	71.02 ± 15.11^d^	220.05 ± 1.96^c^
Do‐CH	0.057 ± 0.028^e^	65.68 ± 9.94^e^	222.74 ± 1.75^b^
Do‐SE	0.047 ± 0.017^e^	67.76 ± 11.14^de^	222.68 ± 2.05^ab^
Do‐S	0.014 ± 0.010^g^	95.87 ± 5.89^b^	222.08 ± 1.77^b^
NE‐NE	0.109 ± 0.0986^d^	79.47 ± 20.28^c^	218.07 ± 2.56^d^
NE‐CH	0.043 ± 0.021^f^	80.22 ± 13.99^c^	221.28 ± 1.77^c^
NE‐SE	0.037 ± 0.019^f^	82.70 ± 13.12^c^	221.35 ± 2.67^c^
NE‐S	0.015 ± 0.009^g^	107.15 ± 8.02^a^	220.95 ± 1.91^c^
CH‐CH	0.145 ± 0.074^c^	56.21 ± 10.71^f^	223.05 ± 1.6^a^
CH‐SE	0.055 ± 0.029^e^	65.00 ± 10.29^d^	223.38 ± 1.98^a^
CH‐S	0.055 ± 0.029^e^	80.29 ± 10.19^c^	222.38 ± 2.35^b^
SE‐SE	0.137 ± 0.100^cd^	59.30 ± 22.87^df^	222.87 ± 1.13^ab^
SE‐S	0.033 ± 0.026^ef^	91.04 ± 13.71^b^	221.79 ± 1.99^bc^
S‐S	0.354^a^	41.18^fg^	213.26^e^

SSF, Seasonal Semideciduous Forest; EDF, Evergreen Dense Forest; EMF, Evergreen Mixed Forest; Do, Lower Doce River Aggregation; NE, Northeastern Aggregation; CH, Central Highland Aggregation; SE, Southeastern Aggregation; and S, Southern Aggregation.

Jaccard similarity as well as phylogenetic turnover computed from both datasets correlate strongly, thus predicting each other (Table 1 from Appendix S3). Furthermore, phylogenetic turnover calculated by the resolved and the unresolved trees shows slopes and coefficients for the correlation near 1, so that improvements to the resolution of angiosperm diversification are expected to alter our results only marginally (Table 2 from Appendix S3).

**Figure 4:**
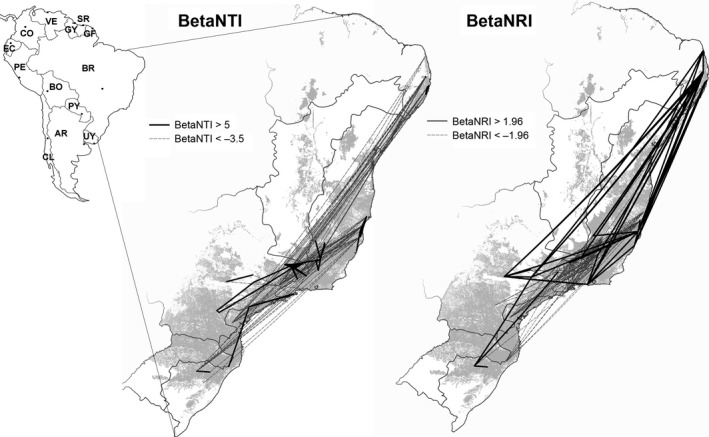
Pairs of communities showing higher or lower phylogenetic turnover than expected by chance in relation to South America. betaNTI is the nearest taxon index, and betaNRI is the net relatedness index from pairs of communities.

**Figure 5:**
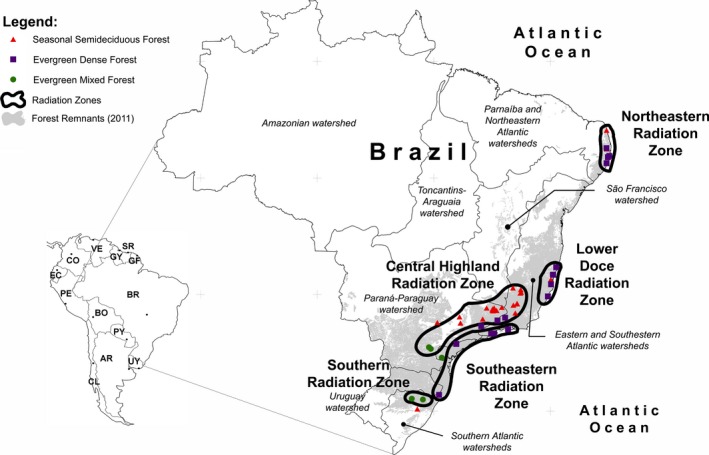
Geographic position of the examined communities in relationship to different Brazilian watersheds and spatial aggregations characterized by coexistence of closely related species.

The significant increase in phylogenetic turnover toward the tip of the phylogenetic tree (betaMNTD) with increases of spatial distance between communities forms a strong spatial pattern. Pairs of communities that show lower phylogenetic turnover toward the tips of the phylogeny than expected by chance are spatially clustered (Figs 4 and 5). betaMNTD within these clusters is lower than between them, while Jaccard similarity shows an opposite tendency (Table 2). In contrast, betaMPD forms no spatial pattern (Figs 4 and 5, Table 2).

Analysis of general linearized mixed models show that Jaccard similarity reduces with spatial distance and environmental differences between study sites; as for Mantel tests, spatial distance explains highest fraction of variance (Table 3). Study sites that are from the same aggrupation defined in Figure 5 show lower turnover than study sites from different aggrupation, which is consistent with results presented in Table 2. betaMNTD shows opposite tendencies, increasing with spatial distance and MAT, but being lower within aggregations than between them. As detected for Mantel tests, spatial distance explains highest fraction of variance. Environmental differences influence betaMPD significantly as do the differences within and between spatial aggregations, but the whole model explains less than 5% of data variance (Table 3).

**Table 3:** Best general linearized mixed models fitting the variables taxonomic similarity (J) and phylogenetic turnover (Log betaMPD and Log betaMNTD) including percentage of explained variance by each variable and overall variance explained by complete model

Variable 1	Variable 2	Fitting parameter	Significance level	Explained variance (*R* ^2^) by variable	Explained variance by model
J	Spatial distance	−0.070	0.001	0.533	0.609
	MAT	−0.006	0.001	0.314	
	MAP	−1.35*10^−5^	0.001	0.035	
	Same spatial aggrupation	0.020	0.001	0.394	
Log betaMNTD	Spatial distance	0.088	0.001	0.376	0.447
	MAT	0.014	0.001	0.276	
	MAP	–	–	–	
	Same spatial aggrupation	−0.020	0.01	0.277	
Log betaMPD	Spatial distance	–	–	–	0.0254
	MAT	2.28*10^−4^	0.001	8.12*10^−5^	
	MAP	1.78*10^−6^	0.001	0.016	
	Same spatial aggrupation	1.45*10^−3^	0.001	0.005	

## Discussion

### Relative importance of dispersal limitation and habitat specialization

Our data indicate that increases in mean annual temperature reduce MPD of angiosperm tree communities, but do not affect their MNTD significantly (Fig. 3). Mean annual precipitation has no significant effect on phylogenetic dispersion. This should be tested more rigorously to allow predictions about phylogenetic diversity and coexistence patterns in the Atlantic Forest under different climate change scenarios. Furthermore, although Duarte et al. (2014) present different findings, the observation of at most weak differences between different habitat types, contradict the interpretation about environmental filtering as well.

Conspecific tree species occur spatially aggregated, as indicated by nonrandom species turnover with spatial distance (Table 1). Phylogenetic turnover measured by betaMNTD increases significantly with spatial distance but lesser with differences in temperature and precipitation regimes (MAT and MAP) between study sites (Tables 1 and 3). betaMPD, on contrast, depends only little on them (Table 3). Lacking phylogenetic resolution and incomplete identification of some taxa do not influence the tendencies revealed in this study (Appendix S3).

The results for similarity indicate dispersal limitation (Chave 2008) or environmental filtering if we assume that environmental differences increase over space (Condit et al. 2002; Fine and Kembel 2011). The latter is consistent with the findings that increasing environmental differences (MAT and MAP) between communities reduce Jaccard similarity (Table 1). Further environmental differences not analyzed here, for example, soil properties such as nutrient and water availability or aluminum toxicity, might reinforce this interpretation.

Such habitat specialization results in the coexistence of closely related species, if ecological niches and functional traits are conserved within evolutionary lineages (e.g., Webb et al. 2002). Assuming increasing environmental differences with spatial distance between communities in combination with phylogenetic signal in trait data, that is, a significant relationship between differences in traits and phylogenetic distances, might therefore be able to explain once the observed spatial pattern of betaMNTD as well as that of Jaccard similarity. Nevertheless, we would expect more significant relations between phylogenetic turnover and environmental differences in such a scenario of environmental filtering than actually observed.

Beside this antagonism resulting from our data, the lack of phylogenetic signal observed in many trait data (Losos 2008; Cavender‐Bares et al. 2009; Godoy et al. 2014) put in question the explanation of habitat specialization as the underlying mechanism causing the observed pattern of taxonomic and phylogenetic turnover. Lacking support for environmental filtering from data and trait conservatism from the literature encourages the adoption of dispersal limitation within the Brazilian Atlantic Forest as the most plausible explication of observed spatial similarity patterns.

### The role of underlying evolutionary processes

The observed spatial turnover of betaMNTD indicates closely related angiosperm tree species to coexist in sympatry (Table 1), while tendencies observed in betaMPD reveal no spatial pattern. Pattern of betaMNTD forms five spatial aggregations in the Atlantic Forest (Fig. 5) characterized by lower phylogenetic turnover between tree communities from the same aggregation than from different ones (Table 3). Nevertheless, differences in mean annual precipitation and temperature between communities increase phylogenetic turnover measured as betaMNTD and reduce Jaccard similarity (Tables 1 and 3), thus indicating that climatic niche of species seems to be conserved within evolutionary lineages. betaMPD, on contrast, is not significantly influenced by environmental differences between study sites.

As betaMNTD values are referred to more recent evolutionary events, considering only the tips of the phylogenetic tree (Cavender‐Bares et al. 2006), the observed pattern of spatial aggregations and sympatry prevails for recently formed taxa only. Older clades, separated from each other by deep evolutionary splits, seem to be widespread within the Atlantic Forest, as indicated by the lacking spatial pattern of betaMPD (Table 3).

Although our data about Atlantic Forest species distribution is far from being complete, the observed coexistence of recently formed, closely related species is consistent with two different evolutionary scenarios. First, they may have evolved in distinct sites (allopatric speciation), with one or all distinct sites being within or outside the limits of the actual Atlantic Forest biome. The removal of the barrier blocking gene flow might cause secondary contact by the superposition of their actual distributional ranges due to dispersion and, eventually, immigration to the Atlantic Forest. Second, they may have evolved in spatial aggregation by sympatric speciation, with their distribution range (still) restricted due to some type of dispersal limitation (Graham and Fine 2008; Fine and Kembel 2011).

Secondary contact of allopatric evolved species (within or outside the analyzed Atlantic Forest) should encompass larger distribution ranges including regions of origin and of secondary contact. This should result in weaker spatial turnover of similarity and phylobetadiversity toward the tip of the phylogenetic tree (betaMNTD) than were actually observed (Fine and Kembel 2011). Sympatric speciation, on contrast, as a hypothetical driving force for the generation of extant species richness and endemism of the Brazilian Atlantic Forest might happen by neutral polyploidization or due to disruptive selection. In both cases, the climatic niche is conserved, thus explaining not only spatial pattern, but phylogenetic turnover with environmental differences between surveyed communities as well.

Therefore, our results indicate a scenario in which a high degree of sympatric speciation produced the richness of extant angiosperm tree species in the Atlantic Forest. Recent findings from molecular analysis for different taxa from further tropical ecosystems (e.g., Naka et al. 2012; Smith et al. 2014) recognize the dominance of sympatric speciation for the formation of tropical biodiversity and encourage our interpretation.

Identified aggregations in which recently evolved taxa coexist in sympatry might correspond to regions where such hypothesized sympatric speciation took place. This interpretation is supported by the observation that the limits of these aggregations coincide with dispersal barriers. These are the *Serra do Mar* between Central Highland, Southern and Southeastern Aggregation as well as the *Serra de Caparaó* and the Southern part of the *Serra de Espinhaço* between Central Highland and Doce River Aggregation. These dispersal barriers might have prevented the arrival of competitors in neighboring regions, thus triggering sympatric speciation (MacArthur and Wilson, 1967).

Furthermore, identified aggregations overlap with centers of endemism for birds, mammals, butterflies and bamboo and other plant species (Carnaval and Moritz 2008; Murray‐Smith et al. 2008; Amorim et al. 2009; Fontoura and Santos 2010; Werneck et al. 2011). This superposition may be a coincidence due to a bias involving incomplete sampling between endemism centers alias clusters identified here. This bias influences species richness or diversity and might alter observed spatial patterns of phylogenetic turnover. It has been highlighted whenever centers of endemism have been postulated. Nevertheless, this superposition indicates that biodiversity and elevated endemism in the Atlantic Forest evolved by sympatric speciation, also more comprehensive approaches are necessary to identify additional centers of endemism as well as regions where sympatric speciation took place.

## Conclusion

Despite some restrictions, that is, incomplete knowledge about actual species distribution, we state that our results are consistent with an evolutionary scenario of sympatric speciation being the dominant pattern that generated outstanding richness and diversity of angiosperm trees in the Atlantic Forest. Dispersal limitation between different clusters that imped the immigration of competitors and niche occupations might have triggered this sympatric speciation; nevertheless, low dispersal allowed older taxa to disperse and become widespread. This spreading masks the effects resulting from eventually past sympatric speciation. As our data are biased by low sampling effort, our hypothesis about sympatric speciation as the dominant generator of angiosperm tree diversity and endemism in the Atlantic Forest should be tested by more comprehensive approaches reducing accentuated restrictions of our considerations.

## Conflict of Interest

None declared.

## Supporting information


**Appendix S1.** Geographic position and characterization of tree communities.
**Appendix S2.** Differences in phylogenetic community structure and between different habitat types.
**Appendix S3.** Influence of lacking phylogenetic resolution and identification on phylogenetic community structure and turnover.
