# Supplementary material for: The hypothesis of sympatric speciation as the dominant generator of endemism in a global hotspot of biodiversity
Source: Ecol Evol. 2015 Oct 26;5(22):5272–83. doi: 10.1002/ece3.1761 (PMC6102518; doi:10.1002/ece3.1761)
Supplement: Supplementary file 1 — Appendix S1. Geographic position and characterization of tree communities. Appendix S2. Differences in phylogenetic community structure and between different habitat types. Appendix S3. Influence of lacking phylogenetic resolution and identification on phylogenetic community structure and turnover. [file ECE3-5-5272-s001.docx]

**Supplementary Information**

Ecography (xxxx) xx, xxx-xxx.

Sympatric Speciation as the dominant Generator of Endemism in a Global Hotspot of Biodiversity

Markus Gastauer^1*^, Amílcar Walter Saporetti-Junior^1^, Luiz Fernando Silva Magnago^1^, Jeannine Cavender-Bares^2^ and João Augusto Alves Meira-Neto^3*^

**Appendix S1** Geographic position and characterization of tree communities

**Appendix S2** Differences in phylogenetic community structure between different habitat types

**Appendix S3** Influence of lacking phylogenetic resolution and identification on phylogenetic community structure and turnover

**Appendix S1**

Geographic position, habitat type, altitude above sea level (ASL), mean annual temperature (T_mean_), amount of mean annual precipitation (Prec), potential evapotranspiration (PET) and characterization of sampling procedures for 49 tree communities in the Atlantic Forest. DBH, minimum diameter at breast height for trees within the surveyed community; SSF, Seasonal Semideciduous Forest; EDF, Evergreen Dense Forest; EMF, Evergreen Mixed Forest. PQ indicates surveys in which the point-centered quarter method was applied as a sampling procedure, the numbers of sampled trees and treelets are indicated in brackets. All other surveys were obtained using square or rectangular plots, where ^c^ indicates contiguous sampling design.

| **Source** | **Coordinates** | **Municipality – State** | **Habitat type** | **ASL** | **T_mean_ [°C]** | **Prec. [mm]** | **DBH**  **[cm]** | **Sample Area [m^2^]** |
| --- | --- | --- | --- | --- | --- | --- | --- | --- |
| Araujo *et al.,* 2005 | 43°10’41’’ W, 20°54’25’’ S | Bras Pires – MG | SSF | 620 | 21.67 | 1272 | 3.2 | 5000^c^ |
| Bertani *et al.,* 2001 | 47°43’05’’ W, 22°26’26’’ S | Ipeúna – SP | SSF | 740 | 20.29 | 1386 | 5 | 7850 |
| Botrel *et al.,* 2002 | 44°55’ W, 21°24’ S | Ingaí – MG | SSF | 1000 | 19.39 | 1529 | 5 | 10000 |
| Campos *et al.,* 2006 | 42°50’50’’ W, 20°47’45’’ S | Vicosa – MG | SSF | 700 | 19.30 | 1221 | 4.8 | 5000 |
| Campos *et al.,* 2011 | 44°49’56’’ W, 23°20’03’’ S | Ubatuba – SP | EDF | 540 | 23.68 | 2513 | 4.8 | 10000^c^ |
| Caravelas (unpublished, 2011) | 39°29’50’’ W, 17°39’39’’ S | Caravelas – BA | EDF | 40 | 24.49 | 1413 | 4.8 | 5000^c^ |
| Carmo & Assis, 2002 | 50°15’25’’ W, 24°39’10’’ S | Tibagi – PR | EMF | 1020 | 18.88 | 1295 | h > 3 m | 5000 |
| Carvalho *et al.,* 2005 | 44°32’20’’ W, 22°13’03’’ S | Bocaina de Minas – MG | EDF | 1520 | 18.22 | 1699 | 5 | 10400 |
| Cestaro, 2002 | 35°11’ W, 5°54’36’’ S | Parnamirim – RN | SSF | 0 | 25.54 | 1370 | 3.2 | PQ (400) |
| Colonetti *et al.,* 2009 | 49°33’ W, 28°36’ S | Siderópolis – SC | EDF | 1040 | 19.09 | 1438 | 5 | 10000^c^ |
| Dalanesi *et al.,* 2004 | 44°59’ W, 21°20’ S | Lavras – MG | SSF | 1060 | 19.39 | 1529 | 5 | 24000 |
| Dias *et al.,* 1998 | 50°25’ W, 24°31’ S | Tibagi – PR | EMF | 740 | 18.88 | 1295 | 5 | 10000^c^ |
| Durigan *et al.,* 2000 | 49°42’05’’ W, 22°24’11’’ S | Gália – SP | SSF | 600 | 21.02 | 1266 | 5 | 6000^c^ |
| Feitos, 2004 | 34°57’ W, 8°6’ S | Recife – PE | EDF | 0 | 25.43 | 2457 | 4.8 | 10000 |
| França & Stehmann, 2004 | 45°55’54’’ W, 22°42’39’’ S | Camanducaia – MG | EDF | 1640 | 18.43 | 1193 | 4.8 | 7500 |
| Gastauer & Meira Neto, 2013 | 42°50’45’’ W, 20°47’46’’ S | Vicosa – MG | SSF | 700 | 19.30 | 1221 | 3.2 | 10000^c^ |
| Gonzaga *et al.,* 2008 | 44°10’15’’ W, 21°05’55’’ S | Tiradentes – MG | SSF | 920 | 19.20 | 1435 | 5 | 9000 |
| Guilherme *et al.,* 2004 | 48°04’ W, 24°14’ S | Sete Barras – SP | EDF | 720 | 21.80 | 2104 | 5 | 19800 |
| Jesus & Rolim, 2005 | 39°56’37’’ W, 19°07’07’’ S | Linhares – ES | EDF | 20 | 23.53 | 1200 | 10 | 400000 |
| Kozera *et al.,* 2006 | 49°19’ W, 25°25’ S | Curitiba – PR | EMF | 920 | 16.46 | 1408 | 3.2 | PQ (1200) |
| Longhi *et al.,* 2008 | 51°25’12’’ W, 29°49’07’’ S | Montenegro – RS | SSF | 40 | 19.47 | 1347 | 4.8 | 18300 |
| Lopes *et al.,* 2002 | 42°33’ W, 19°34’34’’ S | Dionísio – MG | SSF | 300 | 21.17 | 1192 | 4.8 | PQ (800) |
| Magnago *et al.,* 2011 | 40°13’08’’ W, 20°09’09’’ S | Munícipio da Serra – ES | EDF | 100 | 24.21 | 1275 | 4.8 | 6000 |
| Morangon, 2007 | 42°51’46’’ W, 20°47’50’’ S | Vicosa – MG | SSF | 700 | 19.30 | 1221 | 4.8 | 10000 |
| Nascimento *et al.,* 2001 | 51°53’ W, 28°56’ S | Nova Prata – RS | EMF | 480 | 16.67 | 1838 | 9.6 | 10000^c^ |
| Nunes *et al.,* 2003 | 44°57’50’’ W, 21°13’40’’ S | Lavras – MG | SSF | 900 | 19.39 | 1529 | 5 | 50400^c^ |
| Oliveira Filho *et al.,* 2004 | 43°13’ W, 19°26’ S | Itambé do Mato Dentro – MG | SSF | 680 | 19.51 | 1494 | 5 | 7875 |
| Paula *et al.,* 2004 | 42°51’58’’ W, 20°45’19’’ S | Vicosa – MG | SSF | 640 | 19.30 | 1221 | 4.8 | 10000^c^ |
| Pedro Canário I (unpublished, 2011) | 39°47’33’’ W, 18°17’17’’ S | Pedro Canário – ES | EDF | 40 | 23.83 | 1407 | 4.8 | 5000^c^ |
| Pedro Canário II (unpublished, 2011) | 39°45’27’’ W, 18°18’07’’ S | Pedro Canário – ES | EDF | 0 | 23.83 | 1407 | 4.8 | 5000^c^ |
| Peixoto *et al.,* 2005 | 43°37’45’’ W, 22°58’41’’ S | Rio de Janeiro – RJ | EDF | 0 | 23.33 | 1224 | 4.8 | PQ (800) |
| Pinto *et al.,* 2007 | 42°51’41’’ W, 20°47’54’’ S | Vicosa – MG | SSF | 700 | 19.30 | 1221 | 4.8 | 6000^c^ |
| Reserva Capim (unpublished, 2006) | 42°34’50’’ W, 19°52’10’’ S | Dionísio – MG | SSF | 300 | 21.17 | 1192 | 3.2 | 5000^c^ |
| Rocha *et al.,* 2005 | 45°28’17’’ W, 21°09’19’’ S | Coqueiral – MG | SSF | 860 | 19.39 | 1529 | 5 | 10000 |
| Rochedo de Minas (unpublished, 2006) | 43°01’03’’ W, 21°35’07’’ S | Rochedo de Minas – MG | SSF | 500 | 21.39 | 1582 | 3.2 | 5000^c^ |
| Rochelle, 2008 | 45°05’03’’ W, 23°21’59’’ S | Ubatuba – SP | EDF | 1100 | 21.63 | 3062 | 4.8 | 10000^c^ |
| Rodrigues *et al.,* 2003 | 44°55’ W, 21°23’23’’ S | Luminárias – MG | SSF | 920 | 19.39 | 1529 | 5 | 12800 |
| Rondon Neto *et al.,* 2002 | 50°56’08’’ W, 29°00’03’’ S | Caxias do Sul – RS | EMF | 860 | 16.25 | 1915 | 5 | 8000 |
| São Mateus (unpublished, 2011) | 39°58’ W, 18°42’42’’ S | São Mateus – ES | SSF | 60 | 23.80 | 1211 | 4.8 | 5000^c^ |
| Silva & Soares, 2003 | 47°48’ W, 21°55’ S | Sao Carlos – SP | SSF | 680 | 19.63 | 1495 | 5 | 10000^c^ |
| Silva *et al.,* 2003 | 44°50’ W, 21°09’09’’ S | Ibituruma – MG | SSF | 880 | 19.33 | 1910 | 5 | 10400 |
| Silva *et al.,* 2010 | 45°48’30’’ W, 22°06’06’’ S | São Sebastião de Bela Vista – MG | SSF | 900 | 19.33 | 1416 | 5 | 10800 |
| Silva Jr *et al.,* 2008 | 35°03’45’’ W, 8°12’30’’ S | Cabo de Santo Agostinho – PE | EDF | 100 | 24.17 | 1299 | 4.8 | 10000 |
| Souza, 2008 | 42°31’ W, 19°48’ S | Dionísio – MG | SSF | 280 | 21.17 | 1192 | 3.2 | 12000^c^ |
| Teixeira, 2009 | 35°10’41’’ W, 8°43’39’’ S | Tamandaré – PE | EDF | 40 | 24.16 | 2237 | 4.8 | 10500 |
| Valente *et al.,* 2011 | 43°53’ W, 21°59’ S | Rio Preto – MG | EDF | 1200 | 20.15 | 1618 | 3.2 | 7500^c^ |
| Vilela *et al.,* 1995 | 44°37’ W, 21°21’50’’ S | Itutinga – MG | SSF | 900 | 19.33 | 1910 | 5 | 9450 |
| Vitoria, 2009, area I | 35°10’30’’ W, 7°30’12’’ S | Itambé – PE | SSF | 120 | 23.58 | 1404 | 4.8 | 5000 |
| Vitoria, 2009, area II | 35°11’31’’ W, 7°25’54’’ S | Itambé – PE | EDF | 900 | 19.33 | 1910 | 4.8 | 5000 |

**References**

Araujo, F. S., Martins, S. V., Meira Neto, J. A. A., Lani, J. L. and Pires, I. E. 2005. Florística da vegetação arbustivo-arbórea colonizadora de uma área degradada de caulim, em Brás Pires, MG. - Revista Árvore 29: 983-992.

Bertani, D. F, Rodrigues, R. R., Batista, J. L. F. and Shepherd G. J. 2001. Análise temporal da heterogeneidade florística e estrutural em uma floresta ribeirinha. - Revista Brasileira de Botânica 24: 11-23.

Botrel, R. T., Oliveira-Filho, A. T., Rodrigues L. A. and Curi N. 2002. Influência do solo e topografia sobre as variações da composição florística e estrutura da comunidade arbórea-arbustiva de uma floresta estacional semidecidual em Ingaí, MG. - Revista Brasileira de Botânica 25: 195-213.

Campos, E. P., Silva, A. F., Meira Neto, J. A. A. and Martins, S. V. 2006. Florística e estrutura horizontal da vegetação arbórea de uma ravina em um fragmento florestal no município de Viçosa, MG. - Revista Árvore 30: 1045-1054.

Campos, M. C. R., Tamashiro, J. Y., Assis, M. A. and Joly, C. A. 2011. Florística e fitossociologia do componente arbóreo da transição Floresta Ombrófila Densa das Terras Baixas – Floresta Ombrófila Densa Submontana do Núcleo Picinguaba/PESM, Ubatuba, sudeste do Brasil. - Biota Neotropica 11: 1-12.

Carmo, M. R. B. and Assis, M. A. 2002. Caracterização florística e estrutural das florestas naturalmente fragmentadas no Parque Estadual do Guartelá, município de Tibagi, estado do Paraná. - Acta Botanica Brasilica 26: 133-145.

Carvalho, D. A., Oliveira-Filho, A. T., van den Berg, E., Fontes, M. A. L., Vilela, E. A., Marques, J. J. G. M. and Carvalho, W. A. C. 2005. Variações florísticas e estruturais do componente arbóreo de uma floresta ombrófila alto-montana às margens do rio Grande, Bocaina de Minas, MG, Brasil. - Acta Botanica Brasilica 19: 91-101.

Cestaro, L. A. 2002. Fragmentos de Florestas Atlânticas no Rio Grande do Norte: Relações estruturais, florísticas e biogeográficas. Doctorate’s Thesis, Programa de Pós-Graduação em Ecologia e Recursos Naturais, Centro de Ciências Biológicas e de Saúde, Universidade Federal de São Carlos, São Carlos, São Paulo, Brazil.

Colonetti, S., Citadini-Zanette, V. Martins, R., Santos, R., Rocha, E. and Jarenkow, J. A. 2009. Florística e estrutura fitossociológica em floresta ombrófila densa submontana na barragem do rio São Bento, Siderópolis, Estado de Santa Catarina. Acta Scientiarum. - Biological Sciences 31: 397-405.

Dalanesi, P. E., Oliveira-Filho, A. T. and Fontes, M. A. L. 2004. Flora e estrutura do componente arbóreo da floresta do Parque Ecológico Quedas do Rio Bonito, Lavras, MG, e correlações entre a distribuição das espécies e variáveis ambientais. - Acta Botanica Brasilica 18: 737-757.

Dias, M. C., Vieira, A. O. S., Nakajima, J. N., Pimenta, J. A. and Lobo, P. C. 1998. Composição florística e fitossociologia do componente arbóreo das florestas ciliares do rio Iapó, na bacia do rio Tibagi, Tibagi, PR. - Revista Brasileira de Botânica 21: 94-103.

Durigan, G., Franco, G. A. D. C., Saito, M. and Baitello, J. B. 2000. Estrutura e diversidade do componente arbóreo da floresta na Estação Ecológica dos Caetetus, Gália, SP. - Revista Brasileira de Botânica 23: 371-383.

Feitos, A. A. N. 2004. Diversidade de espécies florestais arbóreas associada ao solo em toposseqüência de fragmento da Mata Atlântica de Pernambuco. Master’s Thesis, Programa de Pós-graduação em Ciência do Solo, Departamento de Agronomia, Universidade Federal Rural de Recife, Recife, Brazil.

França, G. S. and Stehmann, J. R. 2004. Composição florística e estrutura do componente arbóreo de uma floresta altimontana no município de Camundacaia, Minas Gerais, Brasil. - Revista Brasileira de Botânica 27: 19-30.

Gastauer, M. and Meira-Neto, J. A. A. 2013. Community dynamics in a species-rich patch of old-growth forest in a global changing scenario. - Acta Botanica Brasilica 27: 270-285.

Gonzaga, A. P. D., Oliveira-Filho, A. T., Machado, E. L. M., Hargreaces, P. and Machado, J. N. M. 2008. Diagnóstico florístico-estrutural do componente arbóreo da floresta da Serra de São José, Tiradentes, MG, Brasil. - Acta Botanica Brasilica 22: 505-520.

Guilherme, F. A. G., Morellato, L. P. C. and Assis, M. A. 2004. Horizontal and vertical tree community structure in a lowland Atlantic Rain Forest, Southeastern Brazil. - Revista Brasileira de Botânica 27: 725-737.

Jesus, R. M. and Rolim, S. G. 2005. Fitossociologia da Mata Atlântica de Tabuleiro. - Boletim Técnico da Sociedade de Investigações Florestais 19: 1-149.

Kozera, C., Dittrich, V. A. O. and Silva, S. M. 2006. Fitossociologia do componente arbóreo de um fragmento de Floresta Ombrófila Mista Montana, Curitiba, PR, BR. - Floresta 36: 225-237.

Longhi, S. J., Brena, D. A., Scipioni, M. C., Giacomolli, L. Z., Deliberali, G., Longhi, R. V. and Mastella, T. 2008. Caracterização fitossociológica de estrato arbóreo em um remanescente de floresta estacional semidecidual, em Montenegro, RS. - Ciência Rural 38: 1630-1638.

Lopes, W. P., Silva, A. F., Souza, A. L. and Meira-Neto, J. A. A. 2002. Estrutura fitossociológica de um trecho de vegetação arbórea no Parque Estadual do Rio Doce - Minas Gerais, Brasil. - Acta Botanica Brasilica 16: 443-456.

Magnago, L. F. S., Simonelli, M., Martins, S. V., Matos, F. A. R. and Demuner, V. G. 2011. Variações estruturais e características edáficas em diferentes estádios sucessionais de Floresta Ciliar de Tabuleiro, ES. - Revista Árvore 35: 445-456.

Morangon, L. C., Soares, J. J., Feliciano, A. L. P., Lins, C. F. L. and Brandão, S. 2007. Estrutura fitossociologica e classificação sucessional do componente arbóreo de um fragment de Floresta Estacional Semidecidual no município de Viçosa, Minas Gerais. - Cerne 13: 208-221.

Nascimento, A. R. T., Longhi, S. J. and Brena, D. A. 2003. Structure and spatial distribution patterns of tree species in a mixed ombrophylous forest sample in Nova Prata, RS. - Ciência Florestal 11: 105-119.

Nunes, Y. R. F., Mendonça, A. V. R., Botezelli, L., Machado, E. L. M. and Oliveira-Filho, A. T. 2003. Variações da fisionomia, diversidade e composição de guildas da comunidade arbórea em um fragmento de Floresta Semidecidual em Lavras, MG. - Acta Botanica Brasilica 17: 213-229.

Oliveira-Filho, A. T., Carvalho, D. A., Vilela, E. A., Curi, N. and Fontes, M. A. L. 2004. Diversity and structure of the tree community of a fragment of tropical secondary forest of the Brazilian Atlantic Forest domain 15 and 40 years after logging. - Revista Brasileira de Botânica 27: 685-701.

Paula, A. L., Silva, A. F., Marco-Jr., P., Santos, F. A. M. and Souza, A. L. 2004. Sucessão ecológica da vegetação arbóres em uma Floresta Estacional Semidecidual, Viçosa, MG, Brasil. - Acta Botanica Brasilica 18: 407-423.

Peixoto, G. L., Martins, S. V., Silva, A. F. and Silva, E. 2005. Estrutura do componente arbóreo de um trecho de Floresta Atlântica na Área de Proteção Ambiental da Serra da Capoeira Grande, Rio de Janeiro, RJ, Brasil. - Acta Botanica Brasilica 19: 539-547.

Pinto, S. I. C., Martins, S. V., Silva, A. G., Barros, N. F., Dias, H. C. T. and Scoss, L. M. 2007. Estrutura do componente arbustivo-arbóreo de dois estádios sucessionais de floresta estacional semidecidual na Reserva Florestal Mata do Paraíso, Viçosa, MG, Brasil. - Revista Árvore 31: 823-833.

Rocha, C. T. V., Carvalho, D. A., Fontes, M. A. L., Oliveira-Filho, A. T., van den Berg, E. and Melo Marques, J. J. G. S. 2005. Comunidade arbórea de um continuum entre floresta paludosa e de encosta em Coqueiral, Minas Gerais, Brasil. - Revista Brasileira de Botânica 28: 203-218.

Rochelle, A. L. C. 2008. Heterogeneidade Ambiental, Diversidade e Estrutura da Comunidade Arbórea de um trecho da Floresta Ombrófila Densa Atlântica. Master’s Thesis, Instituto de Biologia, Universidade Estadual de Campinas, Campinas, São Paulo, Brazil.

Rodrigues, L. A., Carvalho, D. A., Oliveira-Filho, A. T., Botrel, R. T. and Silva, E. A. 2003. Florística e estrutura da comunidade arbórea de um fragmento florestal em Luminárias, MG. - Acta Botanica Brasilica 17: 71-87.

Rondon Neto, R. M., Watzlawick, L. F., Caldeira, M. V. W. and Schoeninger, E. R. 2002. Análise florística e estrutural de um fragmento de floresta ombrófila mista montana, situado em Criúva, RS – Brasil. - Ciência Florestal 12: 29-37.

Silva, L. A. and Soares, J. J. 2003. Composição florística de um fragmento de Floresta Semidecídua no município de São Carlos-SP. - Revista Árvore 27: 647-656.

Silva, V.F., Venturin, N., Oliveira-Filho, A.T., Macedo, R.L.G., Carvalho, W.A.C., van Den Berg, E. 2003. Caracterização estrutural de um fragmento de Floresta Semidecídua no município de Ibituruna, MG. - Cerne 9: 92-106.

Silva, R. K. S., Feliciano, A. L. P., Marangon, L. C. and Lima, R. B. A. 2010. Florística e sucessão ecológica da vegetação arbórea em área de nascente de um fragmento de Mata Atlântica, Pernambuco. - Revista Brasileira de Ciências Agrárias 5: 550-559.

Silva Jr., J. F., Marangon, L. C., Ferreira, R. L. C., Feliciano, A. L. P., Brandão, C. F. L. S. and Alves Jr, F. T. 2008. Fitossociologia do componente arbóreo em um remanescente de Floresta Atlântica no Município do Cabo de Santo Agostinho, PE. - Revista Brasileira de Ciências Agrárias 3: 276-282.

Souza, P. B. 2008. Diversidade florística e atributos pedológicos ao longo de uma encosta com Floresta Estacional Semidecidual Submontana, zona de amortecimento do Parque Estadual do Rio Doce, MG. Doctorate’s Thesis, Departamento de Biologia Vegetal, Universidade Federal de Viçosa, Viçosa, Minas Gerais, Brazil.

Teixeira, L. J. 2009. Fitossociologica e florística do componente arbóreo em toposequência na Reserva Biológica de Saltinho Pernambuco. Master’s Thesis, Programa de Pós-Graduação em Ciências Florestais, Universidade Federal Rural de Pernambuco, Pernambuco, Brazil.

Valente, A. S. M., Garcia, P. O., Salimena, F. R. G. and Oliveira-Filho, A. T. 2011. Composição, estrutura e similaridade florística da Floresta Atlântica, na Serra Negra, Rio Preto – MG. - Rodriguésia 62: 321-340.

Vilela, E. A., Oliveira-Filho, A. T., Carvalho, D. A. and Gavilanes, M. L. 1995. Flora arbustivo-arbórea de um fragmento de mata ciliar no Alto Rio Grande, Itutinga, Minas Gerais. - Acta Botanica Brasilica 9: 87-100.

Vitoria, E. P. 2009. Estrutura da Vegetação arbórea de dois fragmentos florestais na Zona da Mata Norte de Pernambuco. Master’s Thesis, Programa de Pós-Graduação em Ciências Florestais, Universidade Federal Rural de Pernambuco, Recife, Pernambuco, Brazil.

**Appendix S2**

Differences in phylogenetic community structure and between different habitat types


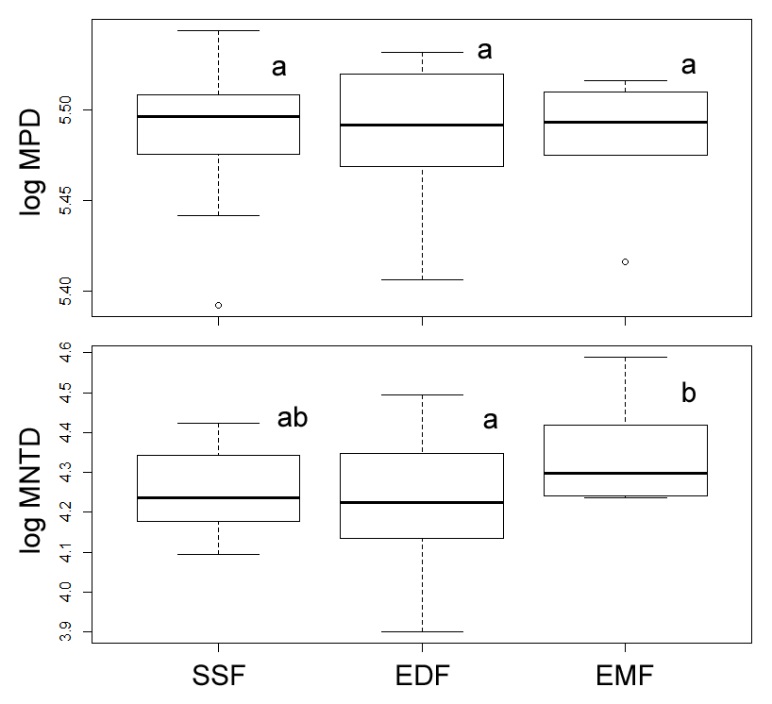


Phylogenetic community structure from 49 tree communities distributed among the three different habitat types Seasonal Semideciduous Forest (SSF), Evergreen Dense Forest (EDF) and Evergreen Mixed Forest (EMF). MPD is the Mean Pairwise Distance, MNTD is the Mean Nearest Neighbor Distance. Different letters indicate significant difference (p < 0.05).

**Appendix S3**

Influence of lacking phylogenetic resolution and identification on phylogenetic community structure and turnover

Table 1. Slopes and r^2^ values from regressing indices of phylogenetic community structure, taxonomic and phylogenetic turnover derived from small dataset on corresponding indices computed with large dataset. See Methods for further explanations.

| **Index** | **Slope** | **r^2^** |
| --- | --- | --- |
| MPD | 0.988 | 0.976 |
| MNTD | 0.993 | 0.987 |
| J | 0.971 | 0.986 |
| betaMPD | 0.995 | 0.981 |
| betaMNTD | 0.993 | 0.998 |

Observations: MPD is the mean pairwise distance, MNTD is the mean nearest-neighbor distance, J is the Jaccard similarity between pairs of communities, betaMPD is the mean pairwise phylogenetic distance from each of two communities, betaMNTD is the mean phylogenetic distance to the closest relative in a paired community.

Table 2. Slopes and r^2^ values from regressing phylogenetic community structure and phylogenetic turnover from unresolved phylogeny built by phylomatic procedure on the same indices computed by randomly resolved phylogenies. The table entries are mean ±standard deviation values from 1000 randomizations.

| **Index** | **Slope** | **r^2^** |
| --- | --- | --- |
| MPD | 0.999 ±0.001 | 0.976 ±0.010 |
| MNTD | 0.976 ±0.013 | 0.915 ±0.017 |
| betaMPD | 0.989 ±0.015 | 0.980 ±0.007 |
| betaMNTD | 0.979 ±0.012 | 0.986 ±0.003 |

Observations: MPD is the mean pairwise distance, MNTD is the mean nearest-neighbor distance, J is the Jaccard similarity between pairs of communities, betaMPD is the mean pairwise phylogenetic distance from each of two communities, betaMNTD is the mean phylogenetic distance to the closest relative in a paired community.
